# The predictive value of the preoperative albumin‐to‐fibrinogen ratio for postoperative hospital length of stay in liver cancer patients

**DOI:** 10.1002/cam4.6606

**Published:** 2023-10-10

**Authors:** Fang Li, Yuetong Ren, Jiacheng Fan, Jin Zhou

**Affiliations:** ^1^ Department of Hepatobiliary Surgery Liaoning Cancer Hospital & Institute, Cancer Hospital of China Medical University Shenyang Liaoning China; ^2^ Department of Medical Laboratory Technology, Medical School Shandong Xiandai University Jinan Shandong China; ^3^ Medical Oncology Department of Gastrointestinal Cancer Liaoning Cancer Hospital & Institute, Cancer Hospital of Dalian University of Technology Liaoning Shenyang China

**Keywords:** albumin‐to‐D‐dimer ratio, albumin‐to‐fibrinogen ratio, hepatocellular carcinoma, postoperative length of stay, preoperative predictive factors

## Abstract

**Background:**

Hepatocellular carcinoma (HCC) is a significant global health burden, with postoperative hospital length of stay (LOS) impacting patient outcomes and healthcare costs. Existing nutritional, inflammatory, and coagulation indices can predict LOS, with particular interest in albumin, fibrinogen, and D‐dimer. This study investigates the predictive value of preoperative albumin‐to‐fibrinogen ratio (AFR) and albumin‐to‐D‐dimer ratio (ADR) for postoperative LOS in HCC patients.

**Methods:**

This retrospective study involved 462 adult HCC patients who underwent partial hepatic lesion excision between February 2016 and August 2022. We analyzed demographic and clinical data, including preoperative blood samples, surgical approach, and LOS. The primary outcome measure was LOS, calculated from the date of surgery to the date of hospital discharge. Preoperative AFR and ADR were calculated. The ROC curves determined optimal cutoff points. The Cox proportional hazards model, Kaplan–Meier method, and the log‐rank test were used for statistical analysis.

**Results:**

The study established an optimal AFR cutoff value of 15.474, with a higher AUC value than ADR, indicating superior predictive potential for postoperative LOS. Participants with high‐AFR (AFR > 15.474) had a shorter median LOS (13 vs. 15 days, *p* < 0.001) compared to those with low‐AFR (AFR ≤15.474). Multivariate analysis revealed high‐AFR (HR: 1.99; *p* < 0.001) as a positive influence on LOS reduction, whereas Child–Pugh rated as B (HR: 0.49; *p* < 0.001), laparotomy (HR: 0.37; *p* < 0.001) and total bilirubin >20.5 μmol/L (HR: 0.58; *p* < 0.001) negatively impacted LOS reduction. Subgroup analysis confirmed AFR's predictive ability for patients experiencing reduced or prolonged LOS due to Child–Pugh score, surgical methods, and total bilirubin concentrations. Even within normal albumin and fibrinogen levels, patients with high‐AFR exhibited a shorter LOS (all *p* < 0.001).

**Conclusions:**

Our findings underscore the value of the AFR as a reliable predictor of LOS in HCC patients. An AFR greater than 15.474 consistently correlated with a shorter LOS, suggesting its potential clinical utility in guiding perioperative management and resource allocation in HCC patients.

## Graphical abstract

AFR, a new predictor for postoperative LOS in HCC, outperforms ADR. High‐AFR (over 15.474) associated with shorter median LOS (13 vs. 15 days). AFR reliably predicts LOS across surgical methods and bilirubin concentrations.

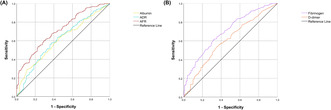

## 1 INTRODUCTION

Hepatocellular carcinoma (HCC) ranks as the fourth principal cause of cancer‐related mortality globally and constitutes the second‐leading contributor to years of life lost due to cancer.^1^ Although hepatectomy serves as the foremost curative intervention for early‐stage HCC, protracted postoperative hospital length of stay (LOS) considerably influences patient outcomes and healthcare expenditures.^2^, ^3^, ^4^ LOS has been employed as a proxy indicator of patient well‐being during hospitalization.^5^ Consequently, minimizing LOS harbors the potential to curtail healthcare costs, mitigate the risk of infections and other nosocomial diseases, and augment patient quality of life.^6^, ^7^ Discerning precise predictors of postoperative LOS prior to surgical intervention is paramount for optimizing perioperative management, diminishing healthcare costs, and enhancing patient prognosis.

Malnutrition, inflammation, and coagulation processes occupy a central role in tumorigenesis, progression, and metastasis, with various nutritional, inflammatory, and coagulation indices exhibiting prognostic potential across diverse malignancies.^8^, ^9^, ^10^, ^11^, ^12^, ^13^ Moreover, patients presenting with malnutrition, inflammation, or hypercoagulability often endure substantially protracted treatment durations, potentially prolonging LOS.^14^, ^15^, ^16^ Among the markers indicative of patients' nutritional, inflammatory, and coagulatory statuses, albumin, fibrinogen, and D‐dimer have garnered increasing interest in recent years. Albumin, both a nutritional and prognostic indicator, bears a strong association with immune and inflammatory responses.^17^, ^18^ Hypoalbuminemia, widely acknowledged to be implicated in adverse postoperative outcomes, may contribute to an extended LOS.^19^, ^20^ Fibrinogen and D‐dimer, coagulation factors, are linked to tumor cell proliferation and metastasis.^21^, ^22^ These factors reflect coagulation and inflammatory activity and share a close relationship with unfavorable postoperative outcomes, potentially leading to an increased LOS.^23^, ^24^, ^25^

The albumin‐to‐fibrinogen ratio (AFR) and the albumin‐to‐D‐dimer ratio (ADR) concurrently represent patients' nutritional, inflammatory, and coagulatory statuses, potentially offering superior efficacy in predicting LOS compared to the individual markers of albumin, fibrinogen, and D‐dimer. AFR and ADR have emerged as potential prognostic indices in an array of diseases, encompassing hepatic disorders.^26^, ^27^, ^28^, ^29^ Nevertheless, the predictive value of AFR and ADR for postoperative LOS in HCC patients warrants further clarification.

This study endeavors to examine the correlation between preoperative AFR, ADR, and postoperative LOS in HCC patients. We postulate that diminished AFR and ADR levels will correlate with extended postoperative hospitalization. By pinpointing patients at elevated risk for protracted LOS, we aspire to contribute to the formulation of tailored perioperative management strategies, ultimately ameliorating patient outcomes and abating healthcare expenditures.

## 2 MATERIALS AND METHODS

### 2.1 Data acquisition

This retrospective investigation received approval from the Ethical Committee of the Liaoning Cancer Hospital & Institute, with a waiver of informed consent. The study encompassed adult subjects who underwent hepatic lesion partial excision at our establishment between February 2016 and August 2022.

### 2.2 Selection of participants

Inclusion criteria stipulated those participants: (1) had an HCC diagnosis confirmed by postoperative histopathology; (2) harbored a neoplastic lesion amenable to complete surgical removal; (3) fell under Child–Pugh classes A and B; (4) experienced <50 mL of blood loss during surgery; and (5) presented with a single tumor focus. Exclusion criteria encompassed patients who (1) were assigned an Eastern Cooperative Oncology Group (ECOG) performance status of 3 or greater; (2) presented with pulmonary infection, severe chronic obstructive pulmonary disease, pulmonary bullae, respiratory or cardiac failure, elevated blood sugar, acute or chronic renal insufficiency, pronounced malnutrition, inflammation, thrombotic disorders, or hepatic failure within a month prior to surgery; (3) were undergoing surgical intervention for disease recurrence; (4) manifested multiple primary malignancies; (5) had undergone neoadjuvant chemotherapy; (6) were admitted to the intensive care unit postoperatively; (7) demonstrated an absence of any preoperative laboratory data; (8) readmitted within 3 months post‐operation due to poor postoperative recovery; and (9) serious postoperative complications occurred, such as wound dehiscence, massive bleeding, serious infection requiring antibiotics, cardiopulmonary failure, cerebral infarction, and pulmonary infarction.

Our study initiated with an extensive pool of 621 patients who were diagnosed with HCC. During the screening process, a significant subset of this population, namely 75 patients, was deemed ineligible for surgical intervention due to their advanced stage of hepatocellular carcinoma. Post this initial screening, a potential candidate pool of 546 patients remained. To ensure the scientific rigor and the reliability of our study, we then applied our stringent patient inclusion and exclusion criteria mentioned above. Following this rigorous refinement process, ultimately, a cohort of 462 surgical patients with HCC were assimilated into the present investigation.

### 2.3 Operational definitions

The determination of postoperative hospital discharge was contingent on the attending surgeon's assessment and conformance to the ensuing hospital discharge criteria: (1) Patient's overall health status is optimal, with restoration of customary dietary and intestinal functionality; (2) successful recuperation from surgical stress, with pain effectively managed; (3) normal body temperature, absence of positive findings upon abdominal examination, and laboratory test outcomes within or near normal parameters; (4) advancement in ambulation, thus enabling daily living activities post‐discharge; (5) absence of wound infection symptoms, with all drains removed; and (6) transition from parenteral to oral therapeutic medications.

The primary outcome measure was the LOS, calculated from the date of surgery to the date of hospital discharge.

### 2.4 Measured variables

Preoperative demographic and clinical data, inclusive of blood samples, age, sex, body mass index (BMI), maximum diameter of tumor (cm), Child–Pugh Class (A or B), surgical approach (either laparotomy or laparoscopy), ECOG score, and LOS, were compiled. Fasting peripheral blood specimens were collected 1–7 days prior to the surgical intervention. The conventional range for BMI is 18.5–23.9 kg/m^2^. Established reference intervals for blood biomarkers encompass: albumin (35–55 g/L), fibrinogen (2–4 g/L), D‐dimer (0–0.5 mg/L), neutrophil counts (2.0–6.3 × 10^9^/L), lymphocyte count (1.1–3.2 × 10^9^/L), monocyte count (0.1–0.6 × 10^9^/L), erythrocyte count (3.8–5.5 × 10^12^/L), hemoglobin (115–150 g/L), platelet counts (100–300 × 10^9^/L), prealbumin (200–400 mg/L), globulin (20–30 g/L), total bilirubin (3.42–20.5 μmol/L), creatinine (CREA, 44–106 μmol/L), and Na^+^ (136–145 mmol/L). The AFR was defined as albumin (g/L) divided by fibrinogen (g/L), and the ADR as albumin (g/L) divided by D‐dimer (mg/L).

### 2.5 Statistical analysis

Continuous variables were expressed as means with standard deviations or median values with interquartile ranges, contingent on distribution normality, and compared utilizing Student's *t* test and the Mann–Whitney *U* test. Categorical variables were presented as frequencies with percentages and examined using the chi‐square test. Receiver operating characteristic (ROC) curves facilitated the calculation of the area under the curve (AUC) and the determination of optimal AFR and ADR cutoff points. The DeLong test was employed to compare ROC curve performance between AFR and ADR. The LOS was analyzed in the Cox proportional hazards model with discharge as outcome event. Hazard ratios (HR) exceeding one signify expedited discharge and a reduced LOS, while HR below one denotes delayed discharge and an extended LOS. Variables exhibiting significance <0.05 in univariable Cox regression analyses were subsequently incorporated into multivariable Cox regression. LOS data were depicted utilizing the Kaplan–Meier method and scrutinized via the log‐rank test with discharge as status variable, where a diminished Kaplan–Meier curve suggests a shorter LOS and an elevated Kaplan–Meier curve implies a longer LOS. A *p*‐value <0.05 was deemed statistically significant for all tests. Data analyses were conducted employing SPSS V.26 statistical software (Chicago, Illinois, USA) and R version 4.1.3 (R Foundation for Statistical Computing, Vienna, Austria).

## 3 RESULTS

### 3.1 The optimal cutoff value for AFR

**FIGURE 1:**
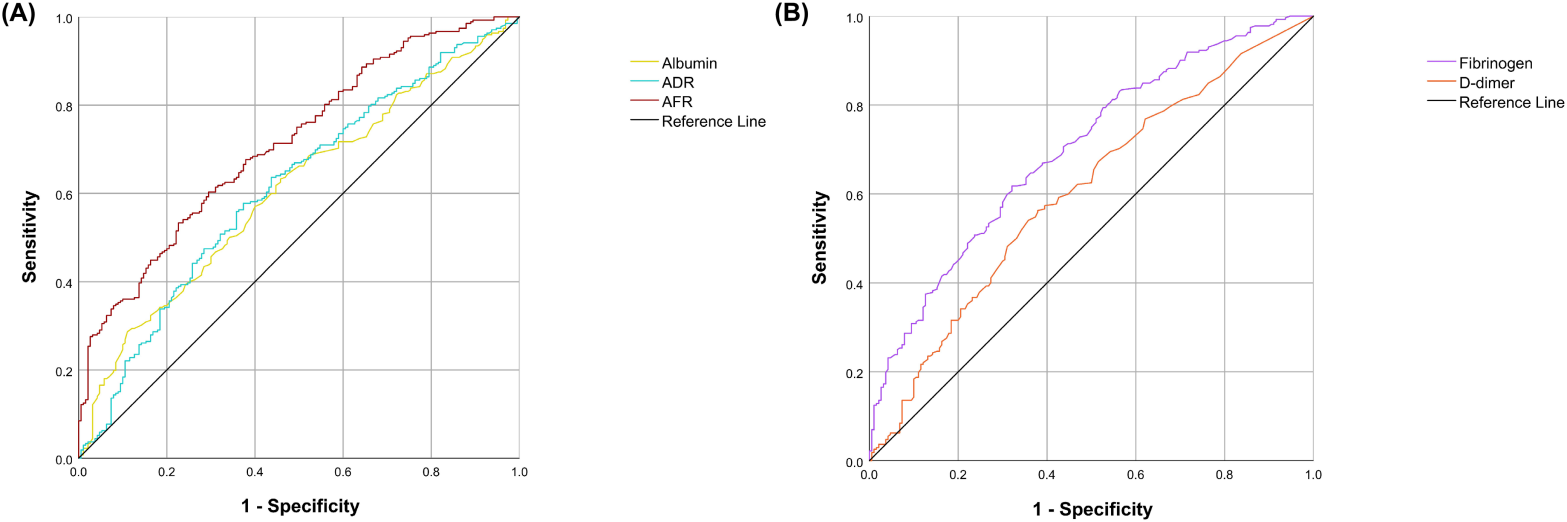
Receiver operating characteristic curves for the ability of preoperative (A) albumin, ADR, AFR and (B) fibrinogen, D‐dimer to predict postoperative hospital LOS for HCC patients. ADR, albumin‐to‐D‐dimer ratio; AFR, albumin‐to‐fibrinogen ratio; HCC, hepatocellular carcinoma; LOS, length of stay.

We employed the continuous variables of albumin, fibrinogen, D‐dimer, AFR, and ADR as test variables, and the median LOS of 14 days as the state variable. An analysis of the ROC curve was conducted to ascertain the AUC value for albumin, fibrinogen, D‐dimer, AFR, and ADR, which were found to be 0.609, 0.699, 0.603, 0.714, and 0.614, respectively. Given that AFR demonstrated a higher AUC value compared to ADR (DeLong test: *p* < 0.001), our ensuing analyses concentrated exclusively on AFR. The analysis indicated an optimal AFR cutoff value of 15.474. Subsequently, participants with AFR > 15.474 were classified into the high‐group, while the remainder were categorized into the low‐group (Figure 1).

### 3.2 Correlation between clinicopathological features and AFR

The sample for this study comprised 75.8% male participants, with a median age of 59 years. We scrutinized the associations between AFR levels and various clinicopathological features of patients. Comparative results revealed that, relative to patients with high‐AFR, those with low‐AFR exhibited an elevated age, augmented levels of neutrophils, monocytes, platelets, and total bilirubin, and diminished levels of red blood cells, hemoglobin, prealbumin, creatinine, and Na^+^ (all *p* < 0.05). Patients exhibiting elevated AFR demonstrated a heightened prevalence of Child–Pugh score of A, in conjunction with a higher incidence of laparoscopic procedures, relative to those with reduced AFR (all *p* < 0.05). There was no difference between the high‐AFR group and the low‐AFR group in the maximum diameter of tumor (*p* = 0.533; Table 1).

**TABLE 1:** Relationship between the pretreatment AFR and clinicopathological features.

	Total	High‐AFR	Low‐AFR	*p*‐value
Total (*n*)	462	242	220	
Sex (*n*)				
Male	350 (75.8%)	191 (78.9%)	159 (72.3%)	0.096
Female	112 (24.2%)	51 (21.1%)	61 (27.7%)	
Age (years, median)	59.00 (52.00–65.00)	57.00 (51.75–63.00)	60.5 (54.00–65.00)	0.004
BMI (kg/m^2^, median)	23.47 (21.70–25.60)	23.67 (21.83–25.89)	23.03 (21.63–25.21)	0.157
ECOG (n)				
0–1	392 (84.8%)	203 (83.9%)	189 (85.9%)	0.544
2	70 (15.2%)	39 (16.1%)	31 (14.1%)	
Diameter of tumor (cm, median)	5.8 (4.0–7.9)	5.9 (4.1–7.9)	5.8 (3.8–7.8)	0.533
Child–Pugh				
A	408 (88.3%)	241 (99.6%)	167 (75.9%)	<0.001
B	54 (11.7%)	1 (0.4%)	53 (24.1%)	
Surgical method				
Laparotomy	391 (84.6%)	186 (76.9%)	205 (93.2%)	<0.001
Laparoscopic	71 (15.4%)	56 (23.1%)	15 (6.8%)	
Neutrophil counts (×10^9^/L, median)	3.37 (2.60–4.42)	2.98 (2.36–3.90)	3.85 (2.93–4.93)	<0.001
Lymphocyte count (×10^9^/L, median)	1.55 (1.17–1.92)	1.58 (1.20–1.94)	1.51 (1.13–1.89)	0.224
Monocyte count (×10^9^/L, median)	0.35 (0.28–0.46)	0.33 (0.26–0.39)	0.39 (0.30–0.52)	<0.001
Erythrocyte count (×10^12^/L, median)	4.56 (4.11–4.91)	4.70 (4.37–4.98)	4.35 (3.96–4.75)	<0.001
Hemoglobin (g/L, median)	143.00 (131.00–156.00)	147.00 (138.00–157.00)	136.00 (124.00–153.00)	<0.001
Platelet counts (×10^9^/L, median)	173.00 (127.50–218.50)	154.00 (116.00–199.00)	196.50 (151.25–244.25)	<0.001
Prealbumin (mg/L, mean)	203.08 ± 64.02	213.98 ± 61.23	191.09 ± 65.02	<0.001
Globulin (g/L, median)	28.60 (25.48–32.63)	28.65 (24.98–32.33)	28.45 (25.73–32.95)	0.495
Total bilirubin (μmol/L, median)	15.21 (11.56–22.87)	14.17 (11.40–18.71)	18.17 (11.63–47.27)	<0.001
CREA (μmol/L, median)	62.80 (52.48–73.53)	64.90 (54.70–74.00)	60.40 (50.00–73.03)	0.018
Na^+^ (mmol/L, median)	141.00 (139.00–142.00)	141.00 (140.00–143.00)	140.00 (138.00–142.00)	<0.001

Abbreviations: AFR, albumin‐to‐fibrinogen ratio; BMI, body mass index; CREA, creatinine; ECOG, eastern cooperative oncology group.

### 3.3 Predictive factors indicate the LOS of patients

Univariate analyses illuminated that AFR, sex, age, ECOG, Child–Pugh, surgical methodology, neutrophil count, monocyte count, erythrocyte count, hemoglobin, platelet count, prealbumin, total bilirubin, CREA, and Na^+^ were predictive indicators of LOS in patients (all *p* < 0.05). There was no correlation between the patient's maximum diameter of tumor and the patient's LOS (*p* = 0.582).

Upon deeper analysis, it was evident that an AFR > 15.474 (HR: 1.86; *p* < 0.001) exerted a positive influence on LOS reduction in the multivariate analysis. Contrarily, Child–Pugh rated as B (HR: 0.49; *p* < 0.001), the adoption of laparotomy as a surgical method (HR: 0.35; *p* < 0.001), and total bilirubin >20.5 μmol/L (HR: 0.72; *p* = 0.012) had a negative impact on LOS reduction in the multivariate analysis (Table 2).

**TABLE 2:** Correlations between LOS and AFR and other clinicopathological factors.

	Univariate analysis			Multivariate analysis		
	Hazard ratio	95% CI	*p*‐value	Hazard ratio	95% CI	*p*‐value
Sex (female)	0.79	0.64–0.98	0.030	1.01	0.80–1.28	0.914
Age (>59 years)	0.79	0.65–0.95	0.011	1.04	0.86–1.26	0.678
BMI (>23.9 kg/m^2^)	1.20	1–1.44	0.055			
ECOG (=2)	1.38	1.07–1.78	0.014	1.16	0.89–1.51	0.286
Diameter of tumor (>5.8 cm)	1.05	0.88–1.26	0.582			
Child–Pugh (B)	0.31	0.23–0.42	<0.001	0.49	0.32–0.73	<0.001
Surgical method (laparotomy)	0.32	0.24–0.41	<0.001	0.35	0.27–0.46	<0.001
Neutrophil count (>6.3 × 10^9^/L)	0.60	0.42–0.85	0.004	1.39	0.89–2.18	0.149
Lymphocyte count (<1.1 × 10^9^/L)	0.98	0.79–1.23	0.885			
Monocyte count (>0.6 × 10^9^/L)	0.66	0.47–0.92	0.016	0.86	0.57–1.29	0.461
Erythrocyte count (<3.8 × 10^12^/L)	0.50	0.38–0.66	<0.001	1.01	0.70–1.44	0.970
Hemoglobin (<115 g/L)	0.49	0.34–0.7	<0.001	0.74	0.47–1.16	0.193
Platelet count (>300 × 10^9^/L)	0.48	0.33–0.71	<0.001	0.78	0.51–1.21	0.275
Prealbumin (<200 mg/L)	0.79	0.65–0.94	0.010	0.91	0.75–1.11	0.353
Globulin (>30 g/L)	0.88	0.68–1.15	0.354			
Total bilirubin (>20.5 μmol/L)	0.43	0.35–0.54	<0.001	0.72	0.55–0.93	0.012
CREA (<44 μmol/L)	0.67	0.5–0.91	0.011	0.93	0.67–1.28	0.647
Na^+^ (<136 mmol/L)	0.36	0.21–0.62	<0.001	0.79	0.44–1.40	0.416
AFR (>15.474)	2.83	2.31–3.47	<0.001	1.86	1.49–2.32	<0.001

Abbreviations: AFR, albumin‐to‐fibrinogen ratio; BMI, body mass index; CREA, creatinine; ECOG, eastern cooperative oncology group.

### 3.4 Analysis of LOS based on AFR

**FIGURE 2:**
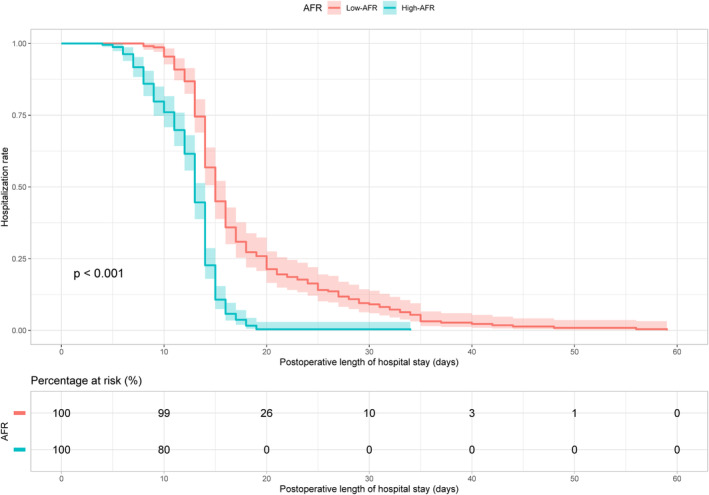
Depiction of Kaplan–Meier plots representing LOS based on the predetermined cutoff value of AFR among the patients. AFR, albumin‐to‐fibrinogen ratio; LOS, length of stay.

The median LOS for the subjects incorporated into this study was established at 14 days. A shorter median LOS was observed in the high‐AFR cohort (13 vs. 15 days, *p* < 0.001) in comparison with their low‐AFR counterparts. As delineated in the accompanying diagram, by the 20th day of LOS, a quarter of the patients within the low‐AFR group remained hospitalized, while complete discharge had been achieved among the high‐AFR group (Figure 2).

### 3.5 Exploratory subgroup analysis of AFR

**FIGURE 3:**
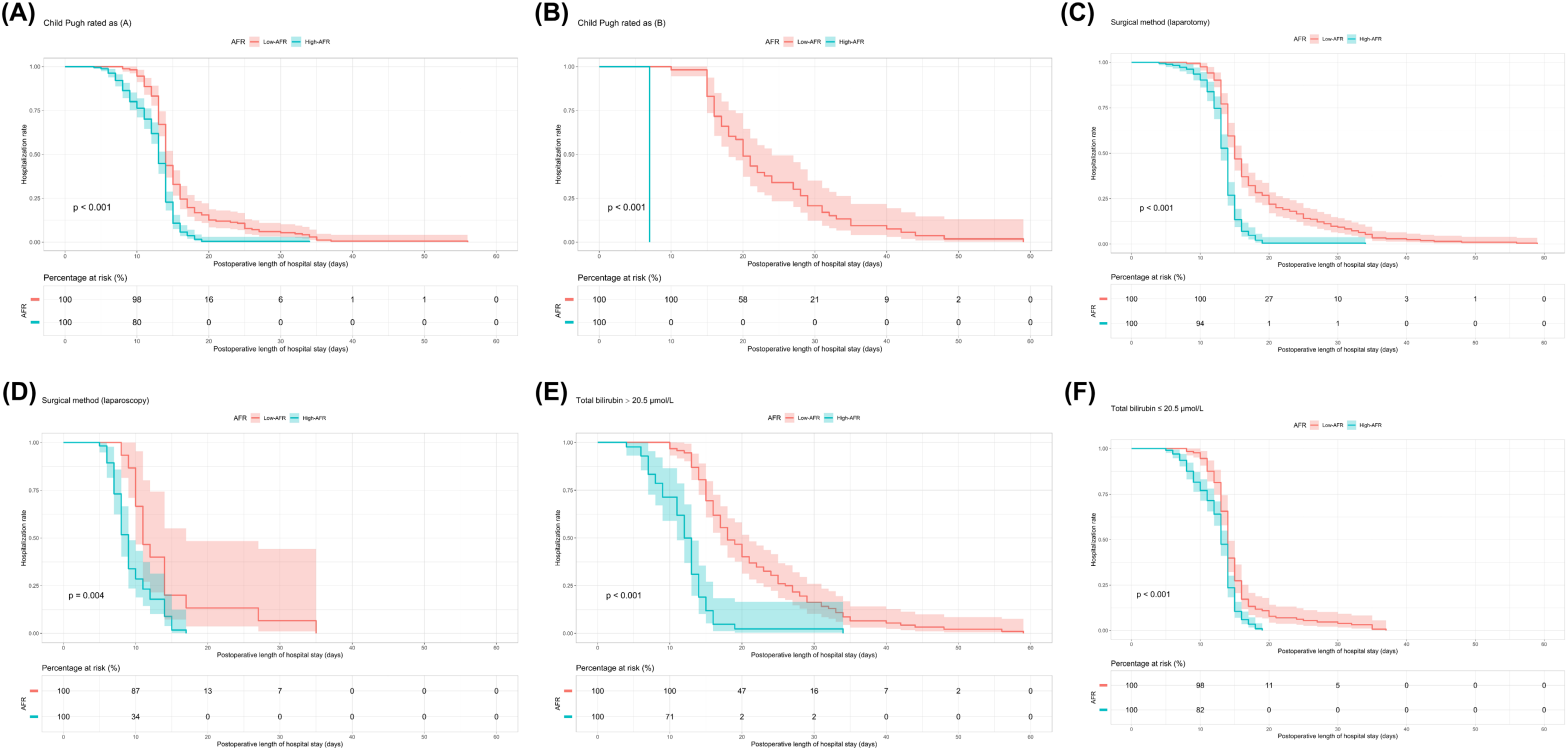
Kaplan–Meier plots of LOS corresponding to the cutoff value of AFR, stratified by (A) Child–Pugh score of A, (B) Child–Pugh score of B, (C) laparotomy surgical method, (D) laparoscopy surgical method, (E) total bilirubin >20.5 μmol/L, and (F) total bilirubin ≤20.5 μmol/L. AFR, albumin‐to‐fibrinogen ratio; LOS, length of stay.

The multivariate analysis presented herein previously identified the Child–Pugh, surgical approach, and total bilirubin levels as influential predictors of LOS. Consequently, we endeavored to determine whether AFR maintains its robust predictive ability for patients experiencing either reduced or prolonged LOS, as influenced by the Child–Pugh, surgical methods, and total bilirubin concentrations. A comparative study revealed that patients within the high‐AFR group experienced a reduction in median LOS, irrespective of the subgroup: patients with a Child–Pugh score of A (13 vs. 14 days, *p* < 0.001, *n* = 408) or Child–Pugh score of B (7 vs. 20 days, *p* < 0.001, *n* = 54), or those undergoing laparotomy (14 vs. 15 days, *p* < 0.001, *n* = 391) or laparoscopic surgery (9 vs. 11 days, *p* = 0.004, *n* = 71), or those characterized by total bilirubin levels either exceeding 20.5 μmol/L (12 vs. 18 days, *p* < 0.001, *n* = 134) or not exceeding 20.5 μmol/L (13 vs. 14 days, *p* < 0.001, *n* = 328) (Figure 3). The cutoff value for total bilirubin, set at 20.5 μmol/L, corresponds to the upper limit of normal in our hospital's laboratory reference values. Therefore, we consider total bilirubin levels exceeding 20.5 μmol/L as abnormal.

**FIGURE 4:**
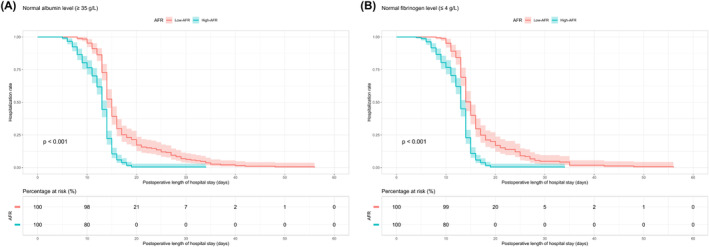
Kaplan–Meier plots of LOS corresponding to the cutoff value of AFR, stratified by (A) normal albumin and (B) normal fibrinogen levels. AFR, albumin‐to‐fibrinogen ratio; LOS, length of stay.

Clinicians may unintentionally overlook patients of potential criticality among those presenting with normal biochemical indices. Given this, our subsequent investigations sought to determine the reliability of AFR as an indicator within the cohort characterized by normal albumin (≥35 g/L) and fibrinogen (≤4 g/L) levels. Our findings underscored that patients with high‐AFR, even within normal albumin (13 vs. 15 days, *p* < 0.001, *n* = 429) and fibrinogen (13 vs. 15 days, *p* < 0.001, *n* = 405) thresholds, exhibited a shorter median LOS in comparison with their low‐AFR counterparts (Figure 4).

## 4 DISCUSSION

Our investigation has yielded substantial results that illuminate the prognostic efficacy of the preoperative AFR in determining the postoperative LOS in patients afflicted with HCC. To our cognizance, our study stands as the pioneer in shedding light on the ramifications of preoperative AFR status on the LOS among surgical patients. The data accrued underscore the potency of AFR as a steadfast and credible metric, equipped to furnish clinicians with indispensable prognostic intelligence.

We delineated the optimal AFR demarcation point to be 15.474, established via meticulous ROC curve analysis. This demarcation served to stratify patients into cohorts exhibiting high‐AFR and low‐AFR. Intriguingly, an elevated AFR was correlated with a reduced LOS, insinuating that an AFR exceeding 15.474 could function as a potential harbinger of a more auspicious postoperative recuperative trajectory. The underpinnings of this association could be rooted in the pivotal role of albumin and fibrinogen in modulating the body's immune response, inflammatory processes, and coagulation dynamics.

Albumin, the principal serum‐binding protein in the human body, executes a myriad of critical physiological functions, including the sustenance of colloidal osmotic pressure, binding of diverse compounds, and furnishing the majority of plasma antioxidant activity.^30^ Albumin can serve as a transport conduit for substances such as bilirubin, fatty acids, metals, ions, hormones, and exogenous pharmaceuticals.^31^, ^32^ Hypoalbuminemia has been validated as a reliable indicator of malnutrition and a prognosticator of mortality in chronic dialysis, acute medical admissions, and septic patients.^33^, ^34^, ^35^ Hypoalbuminemia may also impede postoperative wound healing, compromise patient immunity, augment the risk of infection, and ultimately, precipitate a less favorable postoperative prognosis, and affect the postoperative hospital LOS.^36^, ^37^, ^38^, ^39^

Fibrinogen, a soluble plasma glycoprotein synthesized by the liver, is the antecedent molecule of fibrin, an insoluble protein constituting the scaffold of a blood clot. As a vital coagulation factor, fibrinogen is an indispensable element of the coagulation cascade.^40^ Recent literature has underscored that fibrinogen, functioning as an acute‐phase reactant, surges in response to inflammation, infection, or malignancies.^23^, ^41^ An elevation in fibrinogen often signifies a pro‐thrombotic and pro‐inflammatory state, which invariably impacts the postoperative rehabilitation and prognosis of patients, and extend the postoperative hospital LOS.^42^, ^43^, ^44^

The AFR collectively reflects the inflammatory, nutritional, and coagulation status of patients, potentially proffering superior prognostic precision in postoperative recovery trajectory compared to albumin and fibrinogen considered individually. Our research results also confirmed this. We found that the AUC value of AFR (0.714) was higher than that of albumin (0.609) and fibrinogen (0.699) alone. A heightened AFR may denote a more robust systemic condition and an enhanced capacity to recuperate from surgical stress. Prior research has corroborated that AFR levels can prognosticate postoperative complications in elderly gastric cancer patients subsequent to radical laparoscopic gastrectomy, and overall survival, disease‐free survival, and time to recurrence of patients after curative resection for HCC.^27^, ^45^, ^46^

Our investigation further explored the interplay between clinicopathological characteristics and AFR. It was discerned that individuals with a diminished AFR displayed advanced age, amplified neutrophil, monocyte, platelet, and total bilirubin concentrations, coupled with decreased red blood cell, hemoglobin, prealbumin, creatinine, and Na^+^ levels. These observations intimate that AFR is intimately tethered to a multitude of physiological metrics, potentially serving as a barometer of the patient's overarching health profile.

Moreover, multivariate scrutiny divulged that an augmented AFR, paired with the implementation of Child–Pugh score of B, laparotomy as the surgical strategy and total bilirubin surpassing 20.5 μmol/L, exerted a substantial influence on patient LOS. These inferences suggest that AFR, when harmonized with these variables, might amplify the prognostic acumen for postoperative LOS. The detrimental influence of laparotomy on LOS reduction could be attributable to its inherent invasiveness. Laparotomy, a conventional and more invasive surgical technique, frequently entails an escalated risk of postoperative complications and a protracted convalescence period relative to less invasive surgical modalities such as laparoscopy.^47^, ^48^, ^49^ The correlation of Child–Pugh score of B and total bilirubin concentrations exceeding 20.5 μmol/L with an extended LOS might be linked to its capacity as a sensitive gauge of hepatic function. Elevated Child–Pugh rating and heightened bilirubin levels might denote impaired liver functionality or biliary obstruction, both of which can detrimentally impinge upon the convalescence trajectory post‐HCC surgery.^50^, ^51^, ^52^, ^53^, ^54^

The prowess of AFR as a prognostic instrument was further substantiated through comprehensive subgroup analysis. Irrespective of the surgical modality—encompassing both laparotomy and laparoscopic surgery—and total bilirubin concentrations and Child–Pugh score, patients endowed with an elevated AFR manifested a truncated LOS. This intimates that the predictive efficacy of AFR circumvents alterations in Child–Pugh score, surgical methodology, and bilirubin concentrations, reinforcing its potential as an omnipresent prognostic indicator. Intriguingly, the prognostic merit of AFR remained steadfast even among patients with normative albumin and fibrinogen concentrations. This suggests that AFR could offer pivotal insights in instances where traditional biochemical indices falter.

In summation, our discoveries, for the inaugural time, illuminate the potential of preoperative AFR as a formidable forecaster of postoperative LOS among HCC patients. As a straightforward, economical, and readily available biomarker, AFR could equip clinicians in devising bespoke preoperative and postoperative management tactics, thereby optimizing patient trajectories. While our findings are encouraging, we concede that this study was retrospective in nature, necessitating further explorations to validate the applicability of AFR across varied clinical circumstances and patient demographics. We also advocate for prospective studies to elucidate the potential biological mechanisms intertwining AFR with postoperative LOS in HCC patients. In this study, the variable “operative time” was excluded. While we acknowledge that operative time could influence postoperative hospitalization duration, it is subjected to various confounding factors. Factors such as the surgeon's proficiency, tumor size, and location can significantly influence operative time, hence compromising its objectivity as a standalone measurement. Therefore, it was not included as a variable in this study. Future research could focus on the potential impact of operative time on postoperative outcomes in a more controlled setting, adjusting for these confounding factors. Our study does not explore the potential influence of varying discharge criteria across different medical centers on LOS. Moreover, it is pivotal to underscore that our findings are principally pertinent to a specific cohort: those patients who do not undergo substantial intraoperative blood loss or encounter immediate severe postoperative complications. By focusing on this subset, we aimed to elucidate the pure predictive capacity of AFR in a controlled context. Readers should exercise caution when extrapolating these results to a broader patient population. Therefore, future research could provide a more comprehensive understanding by investigating this aspect, thereby broadening the applicability of our findings and allowing for more nuanced patient management strategies.

## 5 CONCLUSION

This study provides valuable insights into the role of AFR as a robust and reliable indicator of LOS in patients. The results demonstrated that an AFR greater than 15.474 consistently predicted a shorter LOS, indicating a potential utility of AFR as a clinical marker to assist in perioperative patients with HCC management and resource allocation in hospital settings.

## AUTHOR CONTRIBUTIONS

**Fang Li:** Data curation (lead); formal analysis (lead); investigation (lead); methodology (equal); validation (equal); writing – original draft (equal); writing – review and editing (equal). **Yuetong Ren:** Data curation (equal); formal analysis (equal); investigation (equal); methodology (equal); validation (equal); writing – original draft (equal); writing – review and editing (equal). **Jiacheng Fan:** Formal analysis (equal); methodology (equal); validation (equal). **Jin Zhou:** Funding acquisition (lead); project administration (equal); supervision (lead); validation (lead); visualization (lead); writing – review and editing (lead).

## CONFLICT OF INTEREST STATEMENT

The authors have declared that no competing interest exists.

## Data Availability

The data for this study are available from the corresponding author on reasonable request.
